# The fusiform skin paddle in fibula free flap: a fusiform-designed skin paddle for maxillofacial soft defect reconstruction and reducing leg wound tension

**DOI:** 10.3389/fonc.2024.1366079

**Published:** 2024-06-13

**Authors:** Shuai Li, Xin Zheng, Guo-Sheng Cheng, Hua-Ming Mai, Qian-Ting He, An-Xun Wang

**Affiliations:** ^1^ Department of Oral and Maxillofacial Surgery, the First Affiliated Hospital, Sun Yat-Sen University, Guangzhou, China; ^2^ Department of Oral and Maxillofacial Surgery, College of Stomatology, Guangxi Medical University, Nanning, China

**Keywords:** fibula free flap, fusiform, skin graft, closure, complication

## Abstract

**Objective:**

To investigate the feasibility of leg wound closure and reconstruction of maxillofacial soft defect by a fusiform-designed skin paddle in fibula free flap (FFF).

**Methods:**

Fifty patients who underwent FFF for reconstruction of maxillofacial soft defect were divided into two groups. The fusiform group (20 patients) was treated using a fusiform-designed skin paddle in FFF (skin paddle width less than 2 cm), and leg wound was closed using primary suturing. Reconstruction of the maxillofacial soft defect or filling of dead space was achieved by folding the fusiform skin paddle. The conventional group (30 patients) was treated using the conventional-designed skin paddle (skin paddle width no less than 2.5 cm). The leg wound was closed using mattress suturing or skin graft, while reconstruction of the maxillofacial soft defect or filling of dead space by conventional way. The average postoperative length of hospital stay, healing time of leg wound, and post-surgical complications were recorded at least 6 months after the surgery.

**Results:**

Compared with traditional method, the fusiform-designed skin paddle reduced the average healing time of the leg wound (fusiform group: 11.05 days, conventional group: 14.77 days, *P* < 0.05). The average length-to-width ratio in fusiform group was significantly greater than that of in conventional group (fusiform group: 5.85, conventional group: 2.93, *P* < 0.05), and no difference was observed on the graft size of skin paddle between two groups (fusiform group: 23.13, conventional group: 27.13, *P* > 0.05). The post-surgical early complications of the leg wound in the conventional group were higher than that of in the fusiform group (fusiform group: 0%, conventional group: 6.67%), while the post-surgical late complication of the donor site between the two groups showed no case. Healing disorders of maxillofacial soft reconstruction in the conventional group were higher than that of in the fusiform group (fusiform group: 5.26%, conventional group: 20.69%).

**Conclusions:**

Fusiform-designed skin paddle for closure of the leg wound and maxillofacial soft defect is a feasible alternative to the conventional- designed skin paddle. The fusiform- designed skin paddle resulted in the less postoperative length of hospital stay, shorter healing time of leg wound and less complication.

## Introduction

Jaw and soft tissue loss in the maxillofacial area may be caused by several factors, including trauma, tissue atrophy, congenital disease, and malignancies. Its impact extends beyond the effects on morphology, as it also imposes a significant psychological burden on patients. The fibula free flap (FFF) has gained widespread acceptance in the oral reconstructive surgery due to its stable anatomy and satisfactory survival rate following harvest. Additionally, the use of FFF offers the opportunity to address bone and soft tissue deficiencies by with a composite free flap from a single donor site, which is a tremendous advantage over other free flaps (1, 2). To date, FFF is the gold standard and the most reliable method for jaw reconstruction. Its primary benefit lies in its capacity to effectively restore both missing parts of the jaw and associated soft tissue (3).

Despite many efforts, previous studies failed to decrease suture tension of leg wound (2, 4, 5). Many studies have shown that a defect width of leg skin ranging from 3–5 cm is safe. Conversely, instances of leg wound complications have consistently been reported when the defect width of leg skin exceeds 3 cm (6, 7). The tension of the skin on the leg is influenced not only by the defect width, but also by several factors such as nutritional state, age, and development. According to our experiences, it is recommended to limit the acceptable width of skin defects in the legs of young adults to a maximum of 3 cm because of the strength of muscles in this region. If the width of skin paddle is larger than 3 cm, excessive suture tension wound led to several leg wound complications, such as delayed healing, wound dehiscence, and skin necrosis (8–10). In cases of wide skin defect, leg wounds have been closed using either mattress-sutures or skin grafts. The use of skin grafts for the purpose of covering leg wounds has been shown to be associated with extended healing periods and worse aesthetic outcomes.

Therefore, in order to enhance the prognosis of leg wound closure after FFF, it is essential to reduce suture tension of leg wound, without affecting the reconstruction of maxillofacial defect.

## Materials and methods

50 patients with jaw and soft tissue defects who treated in the Department of Oral and Maxillofacial Surgery, the First Affiliated Hospital of Sun Yat-Sen University and College of Stomatology of Guangxi Medical University from January 2017 to March 2024 were enrolled in the study. The clinical data of these patients were retrospectively assessed. The patients were divided into the fusiform group (n = 20) and the conventional group (n = 30). Patients in the fusiform group underwent maxillofacial soft reconstruction using a fusiform skin paddle in FFF, while conventional group received a conventional skin paddle in FFF.

The inclusion criteria were: (1) Patients were suffered from tumors or osteomyelitis; (2) Patients who had underwent jawbone and soft tissue defect after lesion resection in the maxillofacial regions; (3) The patients had no serious systemic disease; (4) There was no injury to the donor leg before operation; (5) Patients with fibula flap reconstruction, and the length of the harvested fibula was longer than 10 cm and less than 20 cm.

All free flaps were performed by surgeons with more than 5 years of experience, and post-operative assessment was identified by two surgeons with more than 5 years’ experience.

### Surgical technique

Preoperative computed tomography angiography was performed to estimate pedal circulation, vessel distribution of the lower limbs, and the risk of post-operative ischemia (11–13). The most prominent contour point of the lateral malleolus and the fibular head was labeled. Then a straight line was drawn to mark the two signs. A handheld ultrasound Doppler was used to assess the precise locations of perforators derived from the peroneal artery and nourish the skin paddle.

### Fusiform- designed skin paddle in FFF (fusiform group)

**Figure 1:**
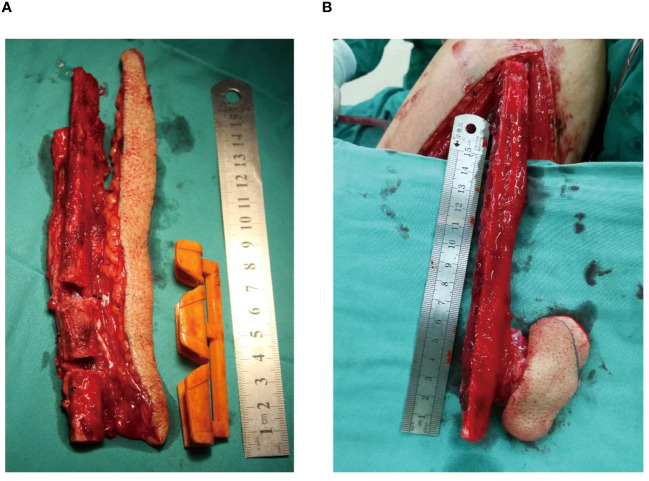
The two different skin paddles. **(A)** The fusiform skin paddle, **(B)** The conventional skin paddle.

**Figure 2:**
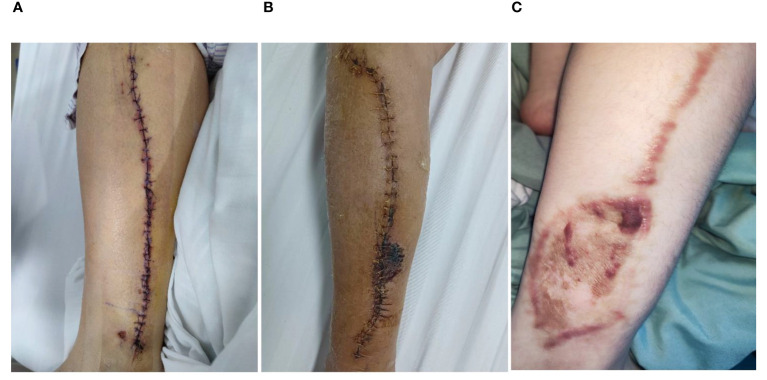
The fibular wound healing after FFF harvesting. **(A)** Under the fusiform skin paddle design, fibular wound was closed using primary suturing, **(B)** Under the conventional skin paddle design, fibular wound was closed by mattress suturing, **(C)** Under the conventional skin paddle design, fibular wound was closed by skin graft.

**Figure 3:**
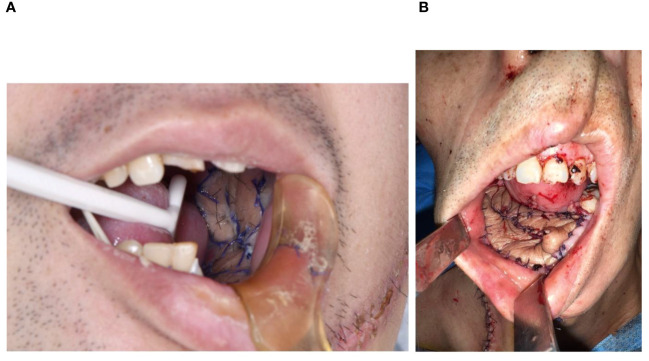
The maxillofacial reconstruction by fusiform skin paddle. **(A)** The defect was reconstructed, and fusiform skin paddle was folded once, **(B)** The defect was reconstructed, and fusiform skin paddle was folded twice.

The maximal width of fusiform skin paddle was less than 2 cm (**Figure 1A**). The fibula, muscle, and vascular pedicle were harvested according to the previous description (14). After the fusiform flap was prepared, the leg wound was closed by primary suturing with a minor tension (**Figure 2A**). The fusiform skin paddle could be folded or de-epithelialized to reconstruct the maxillofacial soft tissue defect and pack dead space (the fusiform skin paddle was folded as an acute angle once or twice, as shown in **Figures 3A, B**).

### Conventional-designed skin paddle in FFF (conventional group)

The fibula, muscle, and vascular pedicle were harvested as previously described (14). Conventional skin paddles (minimal width ≥ 2.5 cm) were designed according to the size of the maxillofacial soft tissue defect without the tension concerns for leg wound closure (**Figure 1B**). After the flap was harvested, the leg wound was closed under mattress-sutures (**Figure 2B**) or skin graft (**Figure 2C**) depending on the tension of leg wound.

### During hospitalization and post-operative assessment

All patients were examined in the outpatient department and telephonically regarding the healing of the donor and recipient sites more than 6 months. In donor site, the occurrence of early complications such as, infection, dehiscence, and local skin necrosis were assessed within 1 month after operation. Late complications such as chronic pain, ankle instability, range of ankle motion, and sensory deficit were observed at least 6 months (10). In recipient site, healing status: infection, fistula, dehiscence and local necrosis was assessed during hospitalization (15). Additionally, the survival rate of flaps, graft size of skin paddle, postoperative length of hospital stay and healing duration of the leg wound were assessed.

### Statistical analysis

All statistical analyses were performed using SPSS version 23.0 (SPSS, Inc., Chicago, IL, USA). Categorical variables were compared between groups using Fisher’s exact test. Continuous variables were expressed as means ± standard deviation (SD), and compared between groups using the student’s t-test to determine significance. For all statistical analyses, two tail *P* < 0.05 was considered to be statistically significant.

## Results

From January 2017 to March 2024, 50 patients underwent jaw and maxillofacial soft tissue reconstruction with FFF were divided into two groups (fusiform group or conventional group), and the flap viability was 96% (2/50). All assessments were completed more than 6 months post-surgery (**Tables 1**–**3**).

**Table 1:** Patients’ demographics and characteristics.

	Fusiform group N=20	Conventional group N=30	*p* value
Age (yr)	49.60 ± 13.76	45.63 ± 14.7	.343
Graft size (cm^2^)	23.13 ± 10.23	27.13 ± 8.93	.149
Length-to-width ratio	5.85 ± 2.46	2.93 ± 0.86	.000
Postoperative hospital stay (day)	12.8 ± 4.01	15.30 ± 5.42	.084
Healing duration of fibula wound (day)	11.05 ± 2.7	14.77 ± 3.34	.000

Symbol ± represents plus or minus sign, reflecting the fluctuation range of the data.

**Table 2:** Postoperative clinical characters and complications at the donor site.

	Early complication		Late complication	
	Fusiform group, N=20	Conventional group, N=30	Fusiform group, N=13	Conventional group, N=30
Cases	0	2 (6.67%)	0	0
*P* value		0.510		–

Symbol - represents no comparison required because no complication occurred between the control and experimental groups.

**Table 3:** Postoperative clinical characters and complications at the recipient site.

	Fusiform group N=19	Conventional group N=29	p value
Healing disorders	1 (5.26%)	6 (20.69%)	0.219

As shown in **Figure 2A**, the leg wound of the fusiform group was primarily closed with minor tension. The skin paddle size ranged from 7.5 cm^2^ to 40 cm^2^ (mean 23.13 cm^2^, the mean length-to-width ratio was 5.85). The maxillofacial soft tissue defects were reconstructed using the fusiform skin paddle. In the conventional group, the leg wound was closed with mattress-sutures or skin graft (**Figures 2B, C**). The flap size range was from 12.5 cm^2^ to 60 cm^2^ (mean 27.13 cm^2^, the mean length-to-width ratio was 2.93). The maxillofacial soft tissue defects were reconstructed using the conventional skin paddle. No difference was observed in graft size between two groups (**Table 1**). The length-to-width ratio was significantly greater in fusiform group than that of in the conventional group (fusiform group: 5.85 ± 2.46, conventional group: 2.93 ± 0.86, *P* < 0.05). The average healing duration of leg wound (fusiform group: 11.05 ± 2.7 days, conventional group: 14.77 ± 3.34 days, *P* < 0.05) and average postoperative length of hospital stay (fusiform group: 12.80 ± 4.01 days, conventional group: 15.30 ± 5.42 days, *P* > 0.05) in the fusiform group were shorter than that of in the conventional group. Skin necrosis of leg wound after closure occurred only in the conventional group (6.67%, **Figures 2A–C**; **Table 2**). Late complications, including chronic pain, ankle instability, range of ankle motion, and sensory deficit, did not occur in either group (**Table 2**). Compared with the conventional group, the fusiform-designed skin paddle enabled the successful coverage of maxillofacial soft defect with folded or de-epithelialized suturing (**Figures 3A, B**; **Table 3**), and the healing complication was less (fusiform group: 5.26%, conventional group: 20.69%).

## Case reports

### Case 1: Floor of mouth defect reconstruction by fusiform skin paddle

**Figure 4:**
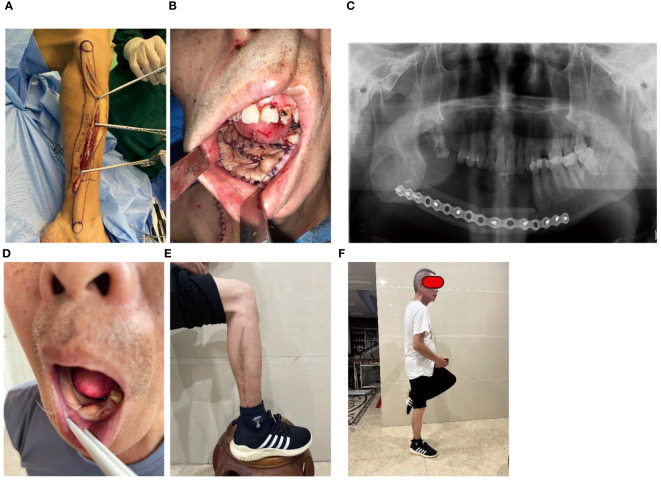
Case 1: Floor of mouth defect reconstruction by fusiform skin paddle. **(A)** The flap was harvested with a fusiform skin paddle, **(B)** The defect was reconstruction by the folded fusiform skin paddle, **(C)** Mandible defect was restored, **(D)** folded fusiform skin paddle healed well, **(E)** Leg healing, **(F)** Standing posture.

A 60-year-old man had a history of gum cancer excision leading to defect of partial mandible and floor of mouth. Physicians harvested a fusiform-designed skin paddle in FFF for reconstruction (**Figure 4A**). The length of the skin paddle was 20 cm, and its width was controlled within 2 cm (**Figure 4A**). Leg wound was closed using primary suturing with a minor tension. The fusiform skin paddle was folded two times to reconstruct floor of mouth (**Figures 4B, C**). Leg wound was healed within14 days, and no healing disorder happened in the recipient site (**Figure 4D**). Late complication was not been observed (**Figures 4E, F**).

### Case 2: Palate defect reconstruction by fusiform skin paddle

**Figure 5:**
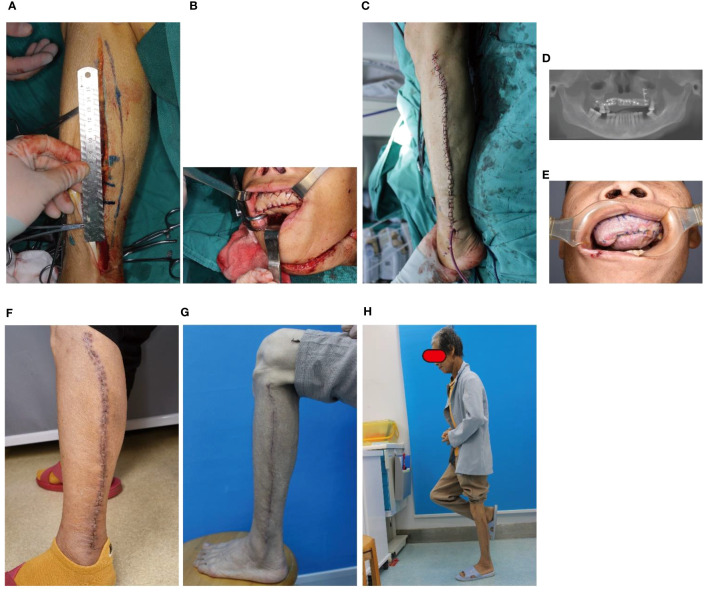
Case 2: Palate defect reconstruction by fusiform skin paddle. **(A)** The flap was harvested with a fusiform skin paddle, **(B)** The defect was reconstruction by the folded fusiform skin paddle, **(C)** The leg wound was closed with a minor tension, **(D)** The defect of partial maxillary was restored by FFF, **(E)** Fusiform skin paddle healed well, **(F, G)** Leg healed well, **(H)** Standing posture.

A 61-year-old woman had a history of gum cancer excision leading to defect of partial maxillary and palate. Physicians harvested a fusiform-designed skin paddle in FFF for reconstruction (**Figures 5A, B**). The length of the skin paddle was 20 cm, and its width was controlled within 2 cm. Leg wound was easily closed (**Figure 5C**). The fusiform skin paddle was folded to reconstruct palate (**Figure 5B**), and the FFF was used to restore the defect of partial maxillary (**Figure 5D**). No healing disorder happened in the recipient site (**Figure 5E**), and leg wound was healed within10 days (**Figure 5F**). Late complication was not been observed (**Figures 5F–H**).

## Discussion

One severe complication caused by radical surgery of oral diseases is jaw and soft tissue defects. The surgical restorations of both the shape and function are clinically challenging. Insufficient reconstruction has the potential to lead to compromised function and an undesired esthetic outcome. In recent decades, there have been notable advancements in the methods and technology used for the reconstruction of composite faults. One of the primary difficulties encountered in reconstructive surgery is the preservation of mandibular continuity, as well as the maintenance of oral mucosal and external skin coverage. Generally speaking, FFF is employed to reconstruct jaw deformities, and the skin paddle in the flap is applied to close the maxillofacial soft tissue defect. The width of the skin paddle in FFF determines the closure technique used at the donor site, and has considerable influence on the subsequent healing process and functional outcomes of the leg after the operation (6).

In our study, the skin paddle was designed in a fusiform shape, enabling for the effective closure of the maxillofacial soft tissue defect (or dead space), as well as facilitating a leg incision with minor stress. The modified flap had a narrow width, measuring around 2 cm, while possessing a substantial length. The fusiform skin paddles can be easily rotated and folded, which resembles a propeller flap (16, 17). In the conventional group, the leg wound, which had a minimum width of 2.5 cm, was closed using mattress sutures or a skin graft. There was no difference observed in the graft size of the skin paddle between the two groups. However, a significant difference was seen in the length-to-width ratio between the two groups. Compared with the conventional group, fusiform design can provide the similar flap size for reconstructive requirement, while the minor tension was beneficial for closure in leg wound.

The average postoperative length of hospital stay and average healing time of the leg wound were reduced in the fusiform group in comparison with the conventional group, which means that the fusiform design in the leg wound was beneficial for leg wound healing. On the other hand, no instances of infection, dehiscence, and local skin necrosis of the leg wound were noted after surgery in the fusiform group, while the conventional group reported 2 cases (6.67%) after treatment of mattress suture or skin graft. We speculated that excessive tension may lead to the local ischemia, resulting in local skin necrosis and prolonging the healing time. No late complication in the leg wound occurred in either group. Consequently, it is reasonable to conclude that both approaches were safe.

The modified skin paddle’s fusiform morphology allows for accommodation of maxillofacial soft tissue defects and dead spaces. In our study, it was observed that the tissue maintained satisfactory shape and exhibited a favorable healing condition, even in instances where the flaps were folded twice or subjected to de-epithelialization (**Figures 3A, B**). The needle puncture test revealed fresh bleeding from the folded flaps, while only one case shown a minor necrosis. So, we speculate that folded skin paddles did not decrease blood circulation. Notably, the flaps were folded to match the maxillofacial tissue defect, thus making the suturing process more difficult. The incidence of injury among the perforators has seen an increase, and risk of poor sealing in the maxillofacial region was elevated. Above all, we recommend that surgeons should pay more attention to protect perforators to enhance the effect of sealing and reconstruction.

While using a fusiform skin paddle with a large length-to-width ratio might effectively reduce closure tension, it is important to acknowledge that this approach may lead to ischemia of the distal tissue of the modified flaps. In order to address this difficulty, it is essential to take the placement of the perforator and the numbers in the flap into account. A median design of perforator and more than one perforator selection can improve the blood supply and decrease the ischemic risk of distal tissue in fusiform skin paddle. In this study, the maximal length-to-width ratio of the fusiform skin paddle was 10, and the largest size was 40 cm^2^. Within the two groups, a size no more than 40 cm^2^ (49 cases within the two groups, accounting for 98% of all cases) can reconstruct most maxillofacial mucous membrane or skin defect. Excessive size was rare which may bring more injures to the leg, and it can be replaced by other flaps.

This study reports the clinical advantages of using a fusiform-shaped skin paddle in FFF. First, the limited width of leg wound can be primarily closed under proper tension. Second, the average postoperative length of hospital stay and the average healing time of the leg wound were reduced. Taken together, these lines of evidence highlight the fusiform-designed skin paddle as an effective alternative to conventional skin paddles.

Nevertheless, there is no denying that our study had several limitations. First, the results of the analyses and conclusions are limited by the small sample size. Second, the shape of mandible reconstructed by fibula was not referred. More high-quality investigations in larger populations are necessary to validate our results in the future.

## Conclusions

The modified FFF described here is a reliable and effective flap for the reconstruction of maxillofacial defects, and the leg wound can be primarily closed under minor tension.

## Data availability statement

The raw data supporting the conclusions of this article will be made available by the authors, without undue reservation.

## Ethics statement

The studies involving humans were approved by the First Affiliated Hospital of Sun Yat-sen University, Clinical Research and Experimental Animal Ethics Committee and the Ethics Committee of School & Hospital, GuangXi Medical University. The studies were conducted in accordance with the local legislation and institutional requirements. Written informed consent for participation was not required from the participants or the participants’ legal guardians/next of kin because The data we collected from our patients did not include critical information about face.Written informed consent was not obtained from the individual(s) for the publication of any potentially identifiable images or data included in this article because As a retrospective study, we applied for exemption from the contents of patients’ informed consent in the process of applying for ethics.

## Author contributions

SL: Data curation, Formal analysis, Writing – original draft, Writing – review & editing. XZ: Data curation, Methodology, Writing – original draft. GC: Data curation, Methodology, Writing – original draft. HM: Formal analysis, Methodology, Writing – review & editing. QH: Formal analysis, Methodology, Writing – review & editing. AW: Supervision, Writing – review & editing.
